# PGT-A in Advanced Maternal Age: The probability of pregnancy is increased?

**DOI:** 10.5935/1518-0557.20250170

**Published:** 2026

**Authors:** M. Valeria Paz, Paula Hovanyecz, Patricia Perfumo, Luciana Domenech, Viviana Ventura

**Affiliations:** 1 Servicio de Medicina Reproductiva, Instituto Gamma - Catamarca, Rosario, Argentina

**Keywords:** preimplantation genetic testing, aneuploidies, embryo biopsy, advanced maternal age

## Abstract

**Objective:**

To determine the probability of achieving a successful pregnancy transferring a euploid embryo per follicular aspiration in women of advanced reproductive age and based on these results to provide an additional clinical tool to support patient counseling in assisted reproductive treatments.

**Methods:**

All patients undergoing follicular aspiration and requiring PGT-A from January 2016 to March 2023 were included. Patients were divided into 4 groups based on age: <35, 35-37, 38-40, and 41 or older. It was calculated for each group the percentage of aspirated cycles with at least one blastocyst for biopsy and the percentage of these cycles with at least one euploid embryo. Ongoing pregnancy rate was calculated for the first euploid embryo transfer. The probability of achieving a successful pregnancy per aspirated cycle was finally determined and it was compared between age groups with Chi-square analysis (significance level of 5%).

**Results:**

There were no significant differences in pregnancy rate per transfer across age groups when euploids embryos were transferred (p=0.61). As patient age increases, the chance of obtaining a blastocyst suitable for biopsy per PGT-A aspirated cycle, and the chance of this embryo being euploid and transferred decrease. Finally, the probability of achieving a successful pregnancy per PGT-A aspirated cycle decreases from 29% in patients younger than 35 years old, to 5% in patients over 40 years old.

**Conclusions:**

Only 5% of women aged 41 or older would achieve pregnancy by transferring a euploid embryo per follicular aspiration. Providing accurate counseling that aligns with the perspective and expectations of these patients will be a challenge for healthcare professionals, even when utilizing the PGT-A technique.

## INTRODUCTION

Maternal age is the strongest predictor of embryo chromosomal abnormalities. As a woman’s age increases, blastocyst euploidy decreases rapidly. Aneuploidy was found to significantly increase with maternal age from 30% in embryos from young women to 70% in women older than 40 years old. The association seems mainly due to chromosomal abnormalities occurring in the oocyte (La Marca *et al*., 2021). Therefore, preimplantation genetic testing for aneuploidies (PGT-A) is considered an essential tool in the selection of euploid embryos for transfer in women of advanced maternal age (AMA) (Practice Committees of the American Society for Reproductive Medicine & the Society for Assisted Reproductive Technology, 2018).

Harton *et al*. (2013) demonstrated, in a multicenter study, that pregnancy and implantation rates when transferring a euploid blastocyst in women of advanced age are comparable to those of younger women, but that the number of cycles without transfers due to the absence of euploids embryos increases in the elderly. In this sense, in that study, the pregnancy rate per biopsied cycle decreased significantly in patients over 40 years old.

To achieve the transfer of a euploid embryo, AMA patients must not only deal with their potentially low ovarian reserve but also with obtaining at least one blastocyst suitable for biopsy. Not to mention that, in order to achieve an embryo transfer, this blastocyst should be chromosomally normal.

The path ahead is not as simple as it seems, given the high expectations of PGT-A in AMA patients. This test does not correct or improve the embryo; it is an additional tool to better select the embryo for transfer. To estimate the real chances of an over-40-year-old woman for achieving a pregnancy by follicular aspiration and based on these results to provide an additional clinical tool to support patient counseling, we conducted this retrospective study at our center.

## MATERIAL AND METHODS

### Study design

Analytical cross-sectional.

### Patients and procedures

This study included all patients, regardless of age, who underwent a follicular aspiration cycle and planned to have chromosomal screening of their embryos from January 2016 to March 2023 at the Servicio de Medicina Reproductiva of the Instituto Gamma. Laboratory procedures, embryo culture, and embryo classification protocols were performed following previously published protocols (Sepúlveda *et al*., 2009; Ebner *et al*., 2001; Paz *et al*., 2017). A single-step individual culture protocol was consistently used, without medium renewal (LifeGlobal or Vitrolife). Embryo biopsies were performed at the blastocyst stage using laser pulses (Lykos, Hamilton Thorne Inc., USA) (Paz *et al*., 2020), and genetic analysis was performed by NGS utilizing Illumina platform.

### Groups of patients and Outcome parameters

Patients were divided into 4 groups according to age: <35, 35-37, 38-40, and 41 years or older.

In each group, the following were calculated: 1) total number of cycles aspirated, 2) percentage of cycles with at least one blastocyst suitable for biopsy (A), 3) percentage of these cycles that had at least one euploid embryo (B), and 4) ongoing pregnancy rate per first euploid embryo transfer (C). The probability of achieving a pregnancy per cycle aspirated (probability of cycles with embryo biopsy (A) x probability of euploid cycles (B) x probability of pregnancy cycles (C) x 100) was estimated.

### Statistical analysis

Clinical outcomes were compared by age group, using the Chi-square test as a statistical analysis, with a significance level of 5%. Aspirations in which the embryos for transfer had less than 60% survival, biopsies and/or very difficult transfers that could have affected the clinical outcome were excluded from the analysis.

## RESULTS

A total of 588 aspirated cycles with a PGT-A indication were analyzed. We found no significant differences in ongoing pregnancy rate after transferring a euploid embryo according to patient age (*p*=0.61). However, a decreasing trend with increasing age was observed (Table 1).

**Table 1:** Results of PGT-A cycles by follicular aspiration and clinical outcomes, according to patient age.

Patient age (years old)	N aspirated cycles	% Cycles with biopsy	% Cycles with euploids embryos	Ongoing pregnancy rate per embryo transfer	Probability of ongoing pregnancy per aspirated cycle
<35	52	73 (38/52)	74 (28/38)	54 (12/22)	29
35-37	72	69 (50/72)	58 (29/50)	61 (14/23)	25^*^
38-40	195	63 (123/195)	41 (50/123)	48 (21/44)	12^*†^
>40	269	43 (116/269)	28 (32/116)	42 (8/19)	5^†^
				*p*=0.61	*p*<0.0001

Values with equal superscripts show significant differences. p^*^=0.004 *p*^†^=0.003.

**Figure 1:**
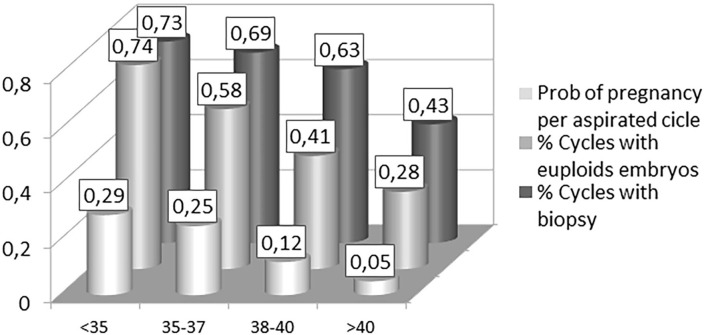
Probability of clinical outcomes per aspirated cycle, according to age group.

When analyzing the results per PGT-A aspiration cycle according to age groups, the final probability of achieving an ongoing pregnancy from aspiration decreases from 29%, in the <35-year-old group, to 5% in patients over 40 years old. As the treatment process advances, the elderly group had 30% fewer chances of obtaining a blastocyst suitable for biopsy compared to the youngest group of patients (43% *vs*. 73%), 45% fewer chances that those biopsied blastocysts were euploid, and approximately 10% fewer chances that the transferred euploid blastocyst would result in an ongoing pregnancy (Figure 1).

## DISCUSSION

Trophectoderm biopsy at the blastocyst stage combined with PGT-A has been gaining widespread acceptance since several studies have shown that PGT-A decreases miscarriage rates, increases implantation rates (Scott *et al*., 2013; Dahdouh *et al*., 2015), and shortens time to pregnancy (Neal *et al*., 2018). However, these authors report pregnancy outcomes based on embryo transfers performed rather than on initiated or aspirated cycles. Munné *et al.* (2019) found no differences in these outcomes for women under 35 years of age, not even when analyzing by initiated cycle.

In women of advanced age, oocyte aneuploidy -and consequently, embryonic aneuploidy- begins to play a significant role. This is why PGT-A is recommended for AMA patients, as an additional tool for embryo selection. In this work, we tried to determine the real chances of an elderly woman for achieving a pregnancy by follicular aspiration. Although the transfer of a euploid embryo in women over 40 years of age results in ongoing pregnancy rates comparable to those in younger women, a non-significant downward trend with increasing maternal age was observed in this study-a 10% difference between the <35 group and those aged 41 or older. Similar results were recently reported by Vitagliano *et al*. (2023), highlighting the importance of continuing to investigate additional age-related factors aside from aneuploidy.

An older woman is also more likely to have a low ovarian reserve, resulting in fewer retrieved oocytes and consequently a lower probability of obtaining a blastocyst suitable for biopsy (Zhu *et al*., 2022). However, the number of usable oocytes is just the first challenge that elderly patients have to face. While the treatment process advances, many other barriers have to be overcome, such as, getting to blastocyst stage, embryo quality suitable for biopsy, embryo euploidy, and these others additional age-related factors mentioned by Vitagliano *et al*. (2023).

From our results we observe that patients over 40 years have 30% less biopsied cycles than <35. Moreover, they have 50% fewer euploid embryos. Thus, the reality faced by these older patients presents a high cost-benefit ratio, challenging the statement that ‘PGT-A shortens time to pregnancy’ as they may have to undergo several cycles of follicular aspiration to obtain a euploid embryo -which must then successfully implant and result in an ongoing pregnancy. Despite the passage of time and scientific evidence, specialists continue to debate how to interpret such findings and how to advise patients in their decision-making regarding PGT-A, taking into account the cost-benefit ratio of the technique.

## CONCLUSION

More prospective studies about this analysis are been necessary for robust conclusion. Nevertheless this restrospective study emphasizes the difficulty faced by patients over 40 years of age in achieving the transfer of a single euploid embryo, concluding that only 5% of women in this age group would achieve pregnancy through follicular aspiration. It remains a challenge for healthcare professionals to provide appropriate counseling from the perspective and expectations of patients of this age, even when utilizing the PGT-A technique.
